# Physics‐guided self‐supervised learning: Demonstration for generalized RF pulse design

**DOI:** 10.1002/mrm.30307

**Published:** 2024-10-09

**Authors:** Albert Jang, Xingxin He, Fang Liu

**Affiliations:** ^1^ Athinoula A. Martinos Center for Biomedical Imaging Massachusetts General Hospital Charlestown Massachusetts USA; ^2^ Harvard Medical School Boston Massachusetts USA

**Keywords:** Bloch equations, deep learning, GPS, online adaptation, RF pulse, self‐supervised learning

## Abstract

**Purpose:**

To introduce a new method for generalized RF pulse design using physics‐guided self‐supervised learning (GPS), which uses the Bloch equations as the guiding physics model.

**Theory and Methods:**

The GPS framework consists of a neural network module and a physics module, where the physics module is a Bloch simulator for MRI applications. For RF pulse design, the neural network module maps an input target profile to an RF pulse, which is subsequently loaded into the physics module. Through the supervision of the physics module, the neural network module designs an RF pulse corresponding to the target profile. GPS was applied to design 1D selective, $B_{1}$‐insensitive, saturation, and multidimensional RF pulses, each conventionally requiring dedicated design algorithms. We further demonstrate our method's flexibility and versatility by compensating for experimental and scanner imperfections through online adaptation.

**Results:**

Both simulations and experiments show that GPS can design a variety of RF pulses with corresponding profiles that agree well with the target input. Despite these verifications, GPS‐designed pulses have unique differences compared to conventional designs, such as achieving $B_{1}$‐insensitivity using different mechanisms and using non‐sampled regions of the conventional design to lower its peak power. Experiments, both ex vivo and in vivo, further verify that it can also be used for online adaptation to correct system imperfections, such as $B_{0}$/$B_{1}^{+}$ inhomogeneity.

**Conclusion:**

This work demonstrates the generalizability, versatility, and flexibility of the GPS method for designing RF pulses and showcases its utility in several applications.

## 1 INTRODUCTION

Deep learning (DL) has profoundly influenced the field of medical imaging. It has been successfully applied in medical image analysis, such as image segmentation, registration, synthesis, and computer‐aided diagnosis.^1^, ^2^ Recently, DL leveraging convolutional neural networks (CNNs) to extract image features using data‐driven approaches has also gained considerable interest and popularity in MRI acquisition and reconstruction. Varying implementations of DL have been shown to enable proficient networks to identify and remove artifacts and noise that arise from undersampled *k*‐space in accelerated MRI.^3^, ^4^, ^5^, ^6^, ^7^, ^8^, ^9^, ^10^, ^11^

On the acquisition side of the MRI process, learning‐based methods have been applied in designing RF pulses. Early studies used a machine learning approach to design RF shimming where models characterize features from simulation data using numerical phantoms and in vivo subjects to achieve patient‐tailored^12^ and calibration‐free^13^ shimming. More recently, DL has also been investigated for RF pulse design. Several pioneering works have used supervised deep learning to generate RF pulses based on learning on the training datasets. Vinding et al.^14^ developed a method to generate multidimensional RF pulses, where a training database was built through Bloch simulation of hundreds of thousands of natural images. This method was further extended to compensate for $B_{0}$/$B_{1}^{+}$ inhomogeneity^15^ with extensively simulated image pairs adding varying $B_{0}$/$B_{1}^{+}$ maps. Zhang et al.^16^ developed a supervised learning approach for designing 2D pulses and their accompanying gradients in a multi‐task fashion through learning simulated image data. Kilic et al.^17^ applied an unsupervised deep learning approach using CNNs with a training database comprised of in vivo $B_{1}^{+}$ maps for static parallel transmit design. Reinforcement learning (RL) is another DL paradigm that uses an agent to act in an environment. Instead of using a traditional data‐driven approach, RL assigns the objective of maximizing a reward based on environmental feedback. Pioneer work has also been shown to use RL in designing RF pulses, where a Bloch simulator was treated as an environment, and the corresponding reward was used to minimize differences between the target RF excitation profile and the estimated profile in design.^18^, ^19^

Although supervised learning and reinforcement learning have both shown great capabilities in designing various RF pulses, they are not without challenges. Supervised learning typically relies on a large amount of labeled data for adequate training, which can be challenging to access in MRI. Although researchers have made creative efforts, such as using extensive natural image databases^14^ and numerical simulations,^12^, ^16^ to augment training data size, there is no guarantee that the trained model will behave optimally when facing severe data discrepancy between training and testing in real applications.^20^ Reinforcement learning is also known to be challenging to implement. Its utilization has been limited because of the non‐trivial design of a suitable state space, action space, and reward function for MRI applications.^21^ Overall, supervised learning and reinforcement learning both require substantial training effort for the model to converge.

Addressing these challenges in supervised learning and reinforcement learning, a new class of self‐supervised learning methods has recently emerged in various MRI applications. Self‐supervised learning requires no labeled data for training, where learning is completed by finding useful feature representation in the input data itself. In the context of MRI reconstruction, MR physics models such as Fourier encoding and coil sensitivity have been used to learn representations of undersampled multi‐coil data to predict unacquired *k*‐space data in self‐supervised learning. Yaman et al.^22^ and Liu et al.^23^ have demonstrated that self‐supervised approaches can perform similarly to their supervised counterparts in MRI reconstruction. Liu et al.^24^ and Bian et al.^25^ additionally incorporated quantitative MR signal models into the self‐supervised learning pipeline to guide the direct generation of quantitative relaxation time maps from undersampled *k*‐space data.

In this study, we introduce a generalized RF pulse design using physics‐guided self‐supervised learning (GPS) method that integrates a physics model into a self‐supervised learning framework as a means of guiding and enforcing the learning process for MRI RF pulse design. The Bloch equation is leveraged as the physics model to enable various RF design applications. In the following, we demonstrate GPS's generalizability by designing 1D selective, $B_{1}$‐insensitive, saturation, and multidimensional RF pulses, each of which conventionally requires separate dedicated design algorithms. By integrating online adaptation in GPS, we further demonstrate the method's flexibility and versatility to compensate for scanner imperfections such as $B_{0}$/$B_{1}^{+}$ inhomogeneity often occurring in real‐time applications.

## 2 THEORY

### 2.1 Supervised learning and self‐supervised learning

Deep learning can facilitate end‐to‐end mapping of input data to a task‐specific domain through learnable parameters.^26^ Supervised learning uses a training library consisting of data/ground truth pairs X/Y with the aim of learning a specific task (i.e., image classification, reconstruction, RF pulse design, etc.). A supervised learning network that takes X as input can be trained by minimizing the following optimization formulation:

(1) $$\hat{\theta }=\underset{\theta }{\text{argmin}}\left(E_{X∼P\left(X\right)}\left[{\left\|D\left(X|\theta \right)-Y\right\|}_{p}\right]\right)$$

where D(X|θ) is a deep learning network with learnable parameters θ that maps input X to Y. $E_{X∼P\left(X\right)}\left[\cdot \right]$ is the expectation operator given X is sampled from the data distribution P(X) for p‐norm loss function ${\left\|\cdot \right\|}_{p}$.

Self‐supervised learning learns to make its own predictions through pretext tasks put forth for its input data. Instead of relying on ground truth labels or preconstructed references, self‐supervised learning generates its own labels or supervisory signals. Training based on these tasks enables the network to learn useful representations of the input data. Its learning process can be formulated as the following:

(2) $$\hat{\theta }=\underset{\theta }{\text{argmin}}\left(E_{X∼P\left(X\right)}\left[{\left\|T\left(D\left(X|\theta \right)\right)-X\right\|}_{p}\right]\right)$$

where T denotes the pretext task incorporated into the framework.

### 2.2 GPS

The pretext task associated with self‐supervised learning can be carried out through the guidance of physics models. The objective function for minimization can be formulated as

(3) $$\hat{\theta }=\underset{\theta }{\text{argmin}}\left(E_{X∼P\left(X\right)}\left[{\left\|P\left(D\left(X|\theta \right)\right)-X\right\|}_{p}\right]\right)$$

where P is the physics model. In MRI RF pulse design, the governing Bloch equations, which describe the dynamics of spins, can naturally serve this purpose. Moving forward, the Bloch equation simulator will be used as the physics model.

### 2.3 Bloch equations

In GPS, the Bloch equations^27^ are simulated to generate supervisory signals used for training. The Bloch equations in the rotating frame of reference in vector form are given by

(4) $$\frac{d}{\mathrm{dt}}\vec{M}=-\gamma {\vec{B}}_{\mathrm{ext}}\left(t\right)\times \vec{M}+\frac{1}{T_{1}}\left(M_{0}-M_{z}\right)\hat{z}-\frac{1}{T_{2}}\left(M_{x}\hat{x}+M_{y}\hat{y}\right)$$

where $\vec{M}=M_{x}\hat{x}+M_{y}\hat{y}+M_{z}\hat{z}$ is the magnetization vector, γ the gyromagnetic ratio, ${\vec{B}}_{\mathrm{ext}}\left(t\right)=c\left(\vec{r}\right)\left(B_{1x}\left(t\right)\hat{x}+B_{1y}\left(t\right)\hat{y}\right)+\left(∆B_{0}\left(\vec{r}\right)+\vec{G}\left(t\right)\cdot \vec{r}\right)\hat{z}$ is any externally applied magnetic field, which includes the time‐varying RF pulse $B_{1x}\left(t\right)\hat{x}+B_{1y}\left(t\right)\hat{y}$ and gradient $\vec{G}\left(t\right)$ along with $c\left(\vec{r}\right)$ and $∆B_{0}\left(\vec{r}\right)$, which reflects any transmit and/or static field inhomogeneity present, $\vec{r}=x\hat{x}+y\hat{y}+z\hat{z}$ denotes position, $M_{0}$ is the equilibrium magnetization and $T_{1}$ and $T_{2}$ are the spin–lattice and spin–spin relaxation times, respectively.

Assuming $T_{1}$ and $T_{2}$ is negligible because its value is typically much greater than the length of the RF pulse, the Bloch equations can be iteratively solved using rotation matrices in a finite small time Δt as

(5) $$\vec{M}\left(t+\Delta t\right)=R\left(t\right)\vec{M}\left(t\right)$$

where the 3 × 3 rotation matrix R(t) is given by

(6) $$R\left(t\right)=R_{z}\left(\phi \left(t\right)\right)R_{y}\left(\beta \left(t\right)\right)R_{x}\left(\alpha \left(t\right)\right)R_{y}\left(-\beta \left(t\right)\right)R_{z}\left(-\phi \left(t\right)\right)$$

Here, $R_{x,y,z}$ are the rotation matrices with respect to the x, y, z axes and α(t), β(t), ϕ(t) its respective rotation amount. In the case when an RF pulse ${\vec{B}}_{1}\left(t\right)$ is applied, ϕ(t) is the RF phase, $\alpha \left(t\right)=2\pi \gamma \left|{\vec{B}}_{\mathrm{eff}}\left(t\right)\right|\Delta t$ is the incremental flip angle for $\left|{\vec{B}}_{\mathrm{eff}}\left(t\right)\right|=\sqrt{{\left|B_{1}\left(t\right)\right|}^{2}+{\left|\Delta B_{0}\left(t\right)\right|}^{2}}$, where $\Delta B_{0}\left(t\right)$ is any resonance offset stemming from gradient fields or $B_{0}$ inhomogeneity and $\beta \left(t\right)=\tan^{-1}\left(\frac{\Delta B_{0}\left(t\right)}{B_{1}\left(t\right)}\right)$.

### 2.4 GPS for RF pulse design

To apply GPS for RF pulse design, the neural network takes a target profile input and maps it to an RF pulse output. The generated RF pulse is then simulated using the Bloch simulator to generate its corresponding profile. The optimization function for GPS RF pulse design becomes

(7) $$\hat{\theta }=\underset{\theta }{\text{argmin}}\left(E_{X∼P\left(X\right)}\left[{\left\|\;P\left(D_{\mathrm{RF}}\left(X_{\mathrm{PF}}^{\mathrm{tgt}}|\theta \right)\right)-X_{\mathrm{PF}}^{\mathrm{tgt}}\right\|}_{p}\right]\right)$$

where $X_{\mathrm{PF}}^{\mathrm{tgt}}$ is the target profile input to the RF pulse design network $D_{\mathrm{RF}}$ and ${\mathrm{RF}}^{*}=D_{\mathrm{RF}}\left(X_{\mathrm{PF}}^{\mathrm{tgt}}|\theta \right)$ is the network‐designed RF pulse. Here, the designed RF pulse is simulated using the Bloch simulator P and its output profile is compared with the target profile $X_{\mathrm{PF}}^{\mathrm{tgt}}$ to form self‐supervision. The objective is to minimize the difference between these two as a self‐supervised loss.

## 3 METHODS

### 3.1 Network architecture and offline learning

**FIGURE 1:**
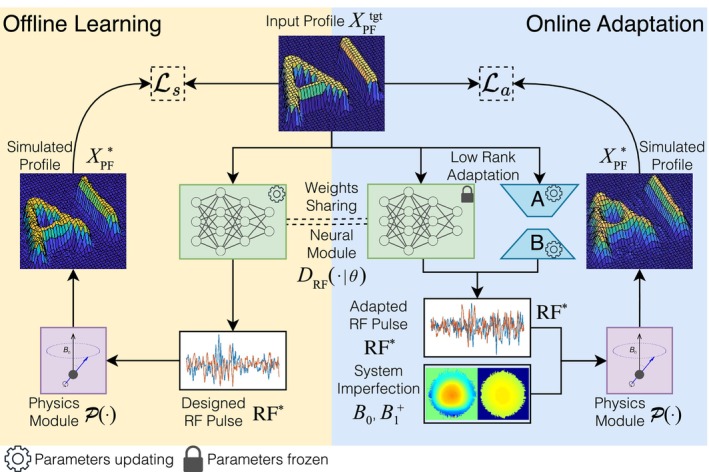
The overall architecture of GPS consists of a neural network module and a physics module. The neural network module takes the target profile $X_{\mathrm{PF}}^{\mathrm{tgt}}$ as input and generates an RF pulse $D_{\mathrm{RF}}\left(X_{\mathrm{PF}}^{\mathrm{tgt}}|\theta \right)$ as output. $D_{\mathrm{RF}}\left(X_{\mathrm{PF}}^{\mathrm{tgt}}|\theta \right)$ is subsequently loaded into the physics module, which outputs the corresponding Bloch‐simulated profile $X_{\mathrm{PF}}^{*}=P\left(D_{\mathrm{RF}}\left(X_{\mathrm{PF}}^{\mathrm{tgt}}|\theta \right)\right)$. The network parameters are trained by minimizing the difference between $X_{\mathrm{PF}}^{\mathrm{tgt}}$ and $X_{\mathrm{PF}}^{*}$. For online adaptation, scanner imperfections such as $B_{0}$/$B_{1}^{+}$ inhomogeneity is included in the physics module. Using the parameters obtained from offline learning as initial values, the neural network module uses methods such as low‐rank adaptation to expedite the learning process.

The overall architecture of GPS consists of a neural network module and a physics module. As stated above and referring to Figure 1 Offline Learning, the neural network module takes target profile $X_{\mathrm{PF}}^{\mathrm{tgt}}$ as input and generates an RF pulse ${\mathrm{RF}}^{*}=D_{\mathrm{RF}}\left(X_{\mathrm{PF}}^{\mathrm{tgt}}|\theta \right)$ as output. The DL network designed RF pulse is subsequently loaded into the physics module, which outputs the corresponding Bloch simulated profile $X_{\mathrm{PF}}^{*}=P\left(D_{\mathrm{RF}}\left(X_{\mathrm{PF}}^{\mathrm{tgt}}|\theta \right)\right)$. The network parameters are trained by minimizing the difference between $X_{\mathrm{PF}}^{\mathrm{tgt}}$ and $X_{\mathrm{PF}}^{*}$ using its mean squared error as a loss function

(8) $$L_{s}=\frac{1}{N}{\left\|\;P\left(D_{\mathrm{RF}}\left(X_{\mathrm{PF}}^{\mathrm{tgt}}|\theta \right)\right)-X_{\mathrm{PF}}^{\mathrm{tgt}}\right\|}_{2}^{2}$$

where N is the number of elements of $X_{\mathrm{PF}}^{\mathrm{tgt}}$.

A feedforward multi‐layer perceptron (MLP) was used as the backbone network for the neural network module of GPS because of MLP's properties of being a universal approximator of any function.^28^ The network consists of two individual subnetworks, each tasked with designing the real and imaginary components of the RF pulse. Each subnetwork includes three sequential fully connected (FC) layers activated using a leaky rectified linear unit. The first input layer takes the vectorized target profile $X_{\mathrm{PF}}^{\mathrm{tgt}}$ with dimension d as input and converts it to a hidden feature vector with dimension d/2. The second and third FC layers further represent the feature vector into a more abstractive feature vector with dimension d/4 followed by the final encoded feature with dimension d/8 (Table 1). The final decoding layer projects the encoded features as the designed RF pulse. In addition, considering that the number of RF pulse elements can vary, the network output dimension was set to a maximum length of 2560 and discarded accordingly during the training process. This provides flexibility with regards to not restricting the number of RF pulse elements (up to 2560) while enabling the design of multidimensional profiles. Furthermore, we define the equilibrium magnetization ($M_{0}$) as 1, which limits the profile range to [−1, 1] and, therefore, eliminates the need to preprocess the input data.

**TABLE 1:** GPS neural network module architecture.

Layer	Input dimension	Output dimension
FC	d	d/2
Leaky ReLU	d/2	d/2
FC	d/2	d/4
Leaky ReLU	d/4	d/4
FC	d/4	d/8
FC	d/8	t

Abbreviations: FC, fully connected; GPS, generalized RF pulse design using physics‐guided self‐supervised learning; ReLU, rectified linear unit.

Notably, instead of using a gigantic database to train a general model for inference, GPS uses a case‐specific training strategy where the network needs to be trained independently for each designed RF pulse. This substantially reduces the need for training data, decreases the training time, and increases design accuracy. Although this “overfitted” model trained under ideal system conditions provides a general baseline for the design target, the flexibility and versatility of our RF pulse design method are realized through rapid online adaptation to real system environments during the scan.

### 3.2 Online adaptation

After offline designing RF pulses for ideal situations, GPS can further adapt the designed RF pulse to compensate for real‐time system imperfections during a scan through online adaptation (Figure 1 Online Adaptation). This is implemented by extending the ideal Bloch simulator P to consider additional system parameters I (e.g., $B_{0}$, $B_{1}^{+}$ inhomogeneity for 2D selective pulse) in the physics module, in which the adaptation loss function can be rewritten as

(9) $$L_{a}=\frac{1}{N}{\left\|\;P\left(D_{\mathrm{RF}}\left(X_{\mathrm{PF}}^{\mathrm{tgt}}|\theta \right),I\right)-X_{\mathrm{PF}}^{\mathrm{tgt}}\right\|}_{2}^{2}$$

In contrast to offline learning, where the network was randomly initialized, the neural network module in online adaptation was initialized with the learned parameters θ transferred from offline learning. Low‐rank parameters for adaptation (LoRA)^29^ were applied to expedite the GPS adaptation process to ensure computational efficiency. LoRA aims to compress a dense network by finding its sparse representation, from where the network can be updated by operating in a small number of network parameters. More specifically, our LoRA implementation fixed the offline learning parameters for MLP to maintain its capability for the specific RF design while fine‐tuning a lightweight side network to quickly adapt to a new training condition by only updating a few low‐rank network parameters.

### 3.3 GPS RF pulse design

To show the generalizability of our method, GPS was demonstrated to design 4 types of RF pulses for different applications, including 1D selective, $B_{1}$‐insensitive, spectral‐spatial selective (SPSP), and multidimensional RF pulses.

#### 3.3.1 One dimensional selective RF pulse

A 1D selective target profile was generated from an RF pulse designed using the Shinnar‐Le Roux (SLR) algorithm^30^ (pulse width, 2.56 ms; time‐bandwidth, 6.6; flip angle, 90°; maximum passband/stopband ripples, 1.5%; least‐squares design). The frequency range of the target profile used for training was [−32.768 kHz, 32.768 kHz].

#### 3.3.2 *B*_1_ ‐insensitive RF pulse

In designing $B_{1}$‐insensitive pulses using GPS, the target profile is 2D, whereby the first dimension is the profile, and the second dimension is the peak amplitude ($B_{1}^{\max}$). A 2D target profile was generated from a hyperbolic secant (HS1) pulse^31^ (pulse width, 8 ms; bandwidth, 2000 Hz), where $B_{1}^{\max}$ was swept from 2.35 to 47 μT in 2.35 μT step increments for a profile frequency range of [−8.192 kHz, 8.192 kHz]. During the design process, the network‐generated RF pulse was normalized to [−1, 1]. The normalized pulse was scaled based on the $B_{1}^{\max}$ values used in the target profile and simulated on the physics module.

#### 3.3.3 Spectral‐spatial selective RF pulse

Like the $B_{1}$‐insensitive case, a 2D target profile is used to design spectral‐spatial selective RF pulses. However, the second dimension of this design is chemical shift. A 2D target profile for this design was generated from a 23.8 ms SPSP pulse^32^, ^33^ with a flip angle of 45°. The pulse consisted of 20 1.19 ms spatial sub‐pulses with spatial time‐bandwidth of 6, whose amplitudes were modulated by a 20‐point spectral envelope with a time‐bandwidth of 3. In anticipation of experiments, the corresponding gradients were designed to meet scanner *G*_max_ and slew rate requirements. Two target profiles were generated: one for water excitation at 0 Hz and another for fat excitation at 440 Hz at 3 T. For both profiles, the first‐dimension profile frequency range was [−50 kHz, 50 kHz], and the second‐dimension chemical shift range was [−750 Hz, 750 Hz].

#### 3.3.4 2D selective RF pulse

To demonstrate GPS's capability of designing arbitrary profiles, a 2D target profile composed of the letters “AI” was used to design a 2D spatial excitation pulse. The target flip angle was set as 15°, and the pulse was generated using an 11.66 ms, 24‐turn variable density spiral‐in trajectory designed to meet scanner gradient specifications.

To assess the performance of the GPS‐designed RF pulse, the Euclidean distance measurement (EDM) for 3D vectors, a metric for measuring the similarity between $X_{\mathrm{PF}}^{\mathrm{tgt}}$ and $X_{\mathrm{PF}}^{*}$, was used, which is defined as

(10) $$\mathrm{EDM}=1-\sqrt{{\left(X_{\mathrm{PF},x}^{*}-X_{\mathrm{PF},x}^{\mathrm{tgt}}\right)}^{2}+{\left(X_{\mathrm{PF},y}^{*}-X_{\mathrm{PF},y}^{\mathrm{tgt}}\right)}^{2}+{\left(X_{\mathrm{PF},z}^{*}-X_{\mathrm{PF},z}^{\mathrm{tgt}}\right)}^{2}}$$

where the subscripts x, y, z denotes the x, y, z components of the vectors $X_{\mathrm{PF}}^{\mathrm{tgt}}$ and $X_{\mathrm{PF}}^{*}$. For $M_{0}$ of 1 used in this study, EDM ranges from [−1,1] where a higher score indicates a closer distance between two magnetic vectors, therefore, better similarity.

### 3.4 Algorithm implementation

The algorithm was programmed using the Python Language with PyTorch package (v. 2.0.0). The Bloch simulator is converted into a C++ module through just‐in‐time (JIT) mechanism^34^ to ensure accelerated computation of matrix rotation operations for each time step of the RF pulse. The MLP uses Kaiming's method^35^ as network initialization for offline learning and AdamW^36^ optimizer to update the learnable parameters for both offline learning and online adaptation. The learning rates were set as 1e^−3^ and 1e^−2^ for offline learning and online adaptation, respectively. The maximum training epochs for offline learning varied depending on the RF pulse type, with the training process concluding once the training loss stopped decreasing while the EDM score reached a predetermined threshold. For 1D selective pulse, $B_{1}$‐insensitive pulse, and SPSP pulse, the threshold for the EDM score was set at 0.999; for 2D selective pulse, it was set at 0.98. As for online adaptation, the maximum fine‐tuning epoch was determined when EDM reached 0.98. All experiments were conducted on a Linux operating system (Rocky Linux release 8.8) equipped with an Intel Xeon Gold 6338 @ 2.00GHz CPU and an NVIDIA A100 GPU.

### 3.5 Experiments

#### 3.5.1 Phantom

Phantom experiments were conducted to verify the GPS pulses. All phantom scans were performed on a Siemens 3 T Skyra system equipped with a 20‐channel head coil.

For the 1D selective and $B_{1}$‐insensitive pulses, its corresponding profiles were measured and compared with its SLR and HS1 counterpart using a modified 1D gradient‐echo sequence on an oil phantom ($T_{1}$/$T_{2}$ = 200/108 ms).^37^ For the 1D selective pulse, the frequency encoding readout direction was taken along the slice direction to project its profile.^38^ Sequence parameters used are TE = 4 ms, TR = 2 s, averages (avg) = 16, 1D matrix size = 256, yielding 0.5 mm resolution along the profile direction. For the $B_{1}$‐insensitive pulse, the sequence required further modification to have it applied as a preparatory module before non‐selective excitation. Using this modification, two separate measurements were carried out, whereby in just one of the two experiments, the $B_{1}$‐insensitive pulse was applied before excitation. The difference between the two measurements was taken to extract the profile. Sequence parameters used are TE = 6 ms, TR = 2 s, avg = 16, $B_{1}^{\max}$ = 2.35–28.2 μT in 2.35 μT step increments, 1D matrix size = 256 yielding 0.5 mm resolution along the profile direction.

The spectral selectivity of the GPS spectral‐spatial selective pulses was compared with its conventional counterpart on a homemade phantom consisting of four 20 mL vials filled with mineral oil ($T_{1}$/$T_{2}$ = 200/108 ms) immersed in a 100 μM MnCl_2_ water bath ($T_{1}$/$T_{2}$ = 1350/86 ms) using a 2D gradient‐echo sequence. Sequence parameters were: 2D matrix size 160 × 160 yielding 1 × 1 mm resolution along the coronal direction, TE/TR = 15/65 ms, and flip angle = 45° using a 5 mm slice thickness. Both target (spectral excitation at 0 and 440 Hz) designed pulses were compared, resulting in four separate measurements.

The excitation profile of the 2D selective pulse was measured on a cylindrical phantom composed of 3.75 g NiSO_4_ × 6H_2_O + 5 g NaCl solution ($T_{1}$/$T_{2}$ = 107/77 ms)^39^ using a 3D gradient‐echo sequence modified to enable 2D RF excitation. Sequence parameters used are 3D matrix size 128 × 128 × 40 yielding 1.3 × 1.3 × 5 mm resolution along the transversal direction, TE/TR = 15/55 ms, and flip angle = 15°. Ramps whose 0^th^ order moment integrated to 0 were added to the beginning of the gradient table to accommodate for scanner's gradient hardware.

#### 3.5.2 In vivo

In vivo experiments were conducted to verify the GPS‐designed RF pulse's applicability in an actual human scan. All in vivo scans were also performed on the same Siemens 3 T Skyra system under a protocol approved by our institution's institutional review board.

A knee scan was conducted to evaluate water and fat imaging using the spectral‐spatial selective pulse. Images using GPS spectral‐spatial selective pulse and its conventional design counterpart were acquired with a 2D gradient echo using a 15‐channel knee coil. Sequence parameters were: 2D matrix size 105 × 105 yielding 1 × 1 mm in‐plane resolution along the knee sagittal direction, TE/TR = 15/60 ms, and flip angle = 45°. Additional anatomical images were acquired using both GPS 1D selective and SLR for comparison and showing anatomical reference. Sequence parameters were: 2D matrix size 105 × 105 yielding 1 × 1 mm in‐plane resolution along the sagittal direction, TE/TR = 5/50 ms, and flip angle = 90°.

A brain scan was conducted to evaluate the 2D excitation profile of the 2D selective pulse. Images using GPS 2D selective pulse for exciting letters “AI” were acquired using the same 3D gradient‐echo sequence and head coil as the phantom, but with parameters: 3D matrix size 128 × 128 × 40 yielding 1.8 × 1.8 × 5 mm resolution along the brain transversal direction, TE/TR = 15/55 ms and flip angle = 15°. An additional image using non‐selective excitation with identical sequence parameters was also acquired for anatomical reference.

#### 3.5.3 Online adaptation

To demonstrate GPS online adaptation to compensate for $B_{0}$/$B_{1}^{+}$ inhomogeneity in 2D selective pulse design for both phantom and brain, the $B_{0}$ and $B_{1}^{+}$ maps were independently measured and incorporated into the Bloch simulator of GPS to fine‐tune and adapt the RF pulse in LoRA using a rank number of 4. The $B_{0}$ maps were obtained using two gradient‐echo images acquired at different echo times (TE1/TE2 = 3/7 ms) and dividing their phase difference map by the difference in echo time,^38^ whereas $B_{1}^{+}$ maps were obtained using the Bloch‐Siegert method.^40^

## 4 RESULTS

Table 2, presented below, provides detailed information on profile dimensions, training iterations, training time, and GPU usage for designing different RF pulse types with GPS.

**TABLE 2:** Detailed information on profile dimensions, training iterations, training time, and GPU usage for designing different RF pulse types with GPS in both offline learning and online adaptation.

RF pulse type	Target profile dimension	No. of RF pulse elements	Iterations	Training time	GPU memory usage (MB)	EDM
Offline learning						
1D selective	2048	256	114	5 min 53 s	1846	0.9996
*B*_1_‐insensitive	2048 × 20	128	302	10 min 33 s	3566	0.9995
SPSP	192 × 96	2453	111	33 min 1 s	17368	0.9995
2D selective	64 × 64	1166	606	2 h 8 min	6718	0.9895
Online adaptation						
2D selective (phantom)	64 × 64	1166	20	4 min 4 s	5282	0.9856
2D selective (in vivo)	64 × 64	1166	20	4 min 2 s	5282	0.9832

Abbreviations: EDM, Euclidean distance measurement; GPS, generalized RF pulse design using physics‐guided self‐supervised learning; GPU, graphics processing unit; MB, megabyte.

### 4.1 GPS RF pulse design (simulation)

#### 4.1.1 One dimensional selective RF pulse

**FIGURE 2:**
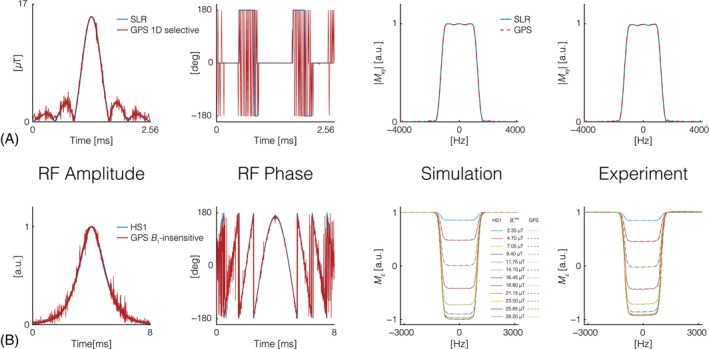
(A) One dimensional selective RF pulse amplitude and phase designed using the Shinnar‐Le Roux (SLR) algorithm (blue) overlaid with generalized RF pulse design using physics‐guided self‐supervised learning (GPS) design (red) are shown on the left. Both simulation and phantom experiment results, displayed on the right half, exhibit that the profiles generated by both pulses agree well. (B) Amplitude and phase modulation functions of the HS1 pulse (blue) overlaid with GPS‐designed $B_{1}$‐insensitive pulse (red) are displayed on the left half. Inversion profiles obtained from sweeping $B_{1}^{\max}$ from 2.35 to 28.2 μT in both simulations and phantom experiments (right half) show excellent agreement in addition to showing $B_{1}$ insensitivity for $B_{1}^{\max}$ values beyond 18.8 μT.

GPS‐designed 1D selective RF pulse overlaid with its SLR counterpart is shown in Figure 2A. Overall, both designed pulses show good agreement, with the GPS design being less smooth, but with a slightly lower peak amplitude (15.15 vs. 15.22 μT). The simulated profile generated using both pulses shows excellent agreement.

**FIGURE 3:**
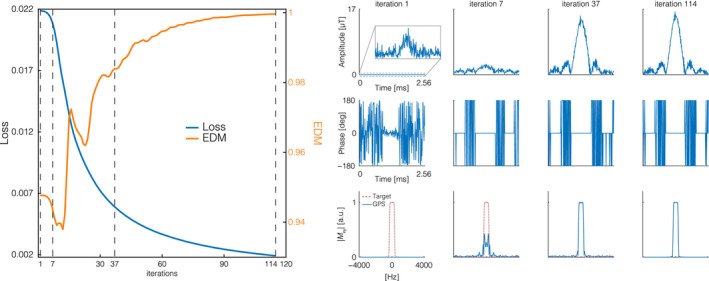
The learning dynamics of the generalized RF pulse design using physics‐guided self‐supervised learning (GPS) framework using 1D selective pulse design as an example. After the first iteration, the RF pulse already starts to take a single‐lobe Gaussian‐like shape. Through the supervision of the physics module, the neural network module learns to design the side lobes, resulting in a profile selectivity that starts to resemble the target (iteration 7). Further down, the overall and relative amplitudes of the 5 lobes are learned (iteration 37), which produces a profile that closely matches the target. In the latter stages of the design process, the pulse is fine‐tuned so that its output optimally matches its target (iteration 114), which describes the low rate of the loss curve's decrease and Euclidean distance measurement (EDM) curve's increase.

Further, an example of the design dynamics for the 1D selective pulse is provided in Figure 3. After the first iteration, the neural network module output RF pulse is noise‐like with low amplitude and a corresponding low amplitude noise‐like excitation profile. Zooming in, however, it is evident that the RF pulse already starts to take a single‐lobe Gaussian‐like shape. As the design process progresses, through the supervision of the physics module, the neural network module learns to design the side lobes, resulting in a profile selectivity that starts to resemble the target (7^th^ iteration). Further down (37^th^ iteration), the overall and relative amplitudes of the 5 lobes are learned, which produces a profile that closely matches the target. In the latter stages of the design process, the pulse is fine‐tuned so that its output optimally matches its target (114^th^ iteration), which describes the low rate of decrease in the loss curve.

#### 4.1.2 *B*_1_ ‐insensitive RF pulse

GPS‐designed $B_{1}$‐insensitive RF pulse overlaid with its HS1 counterpart is shown in Figure 2B. Similar to the 1D selective case, both designed pulses show relatively good agreement, with the GPS design being less smooth. However, the fluctuation of its phase becomes more pronounced at the beginning and end of the pulse, where the amplitude is relatively low. Despite this, the simulation results of both pulses show excellent agreement in terms of both profiles at various $B_{1}^{\max}$ values and $B_{1}$ insensitivity for $B_{1}^{\max}$ values beyond 18.8 μT.

#### 4.1.3 SPSP selective RF pulse

**FIGURE 4:**
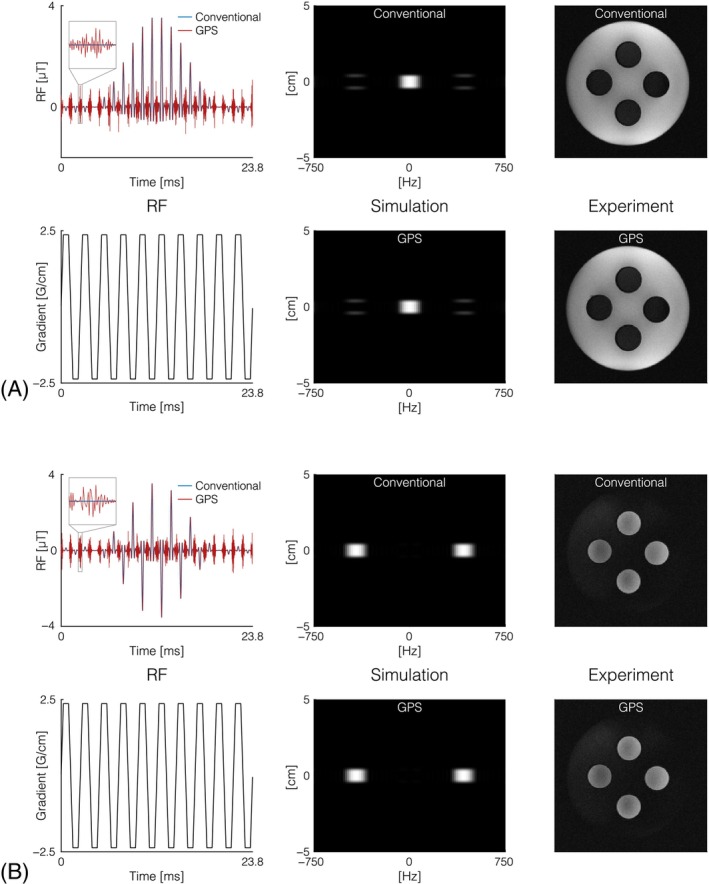
(A) 0 Hz spectral excitation spectral‐spatial (SPSP) pulse designed using conventional method (blue) overlaid with generalized RF pulse design using physics‐guided self‐supervised learning (GPS) design (red) is displayed on the left column. As shown in the zoom‐in, in the gradient ramp regions, the pulse is 0 in the conventional design, whereas the GPS design is not. Despite these differences, simulation results obtained using both pulses (center column) show good agreement in terms of spectral and spatial selectivity. This was further confirmed through phantom experiments (right column). (B) 440 Hz spectral excitation SPSP pulse designed using the conventional method (blue) overlaid with GPS design (red) is shown on the left column. Similar to the 0 Hz case, the GPS design differs from the conventional design in that it is not 0 in gradient ramp regions (zoom‐in). The simulation (center column) and phantom experiment (right column) results obtained from both pulses agree well.

Superimposed GPS‐designed and conventional design SPSP pulses are shown in Figure 4 for both 0 and 440 Hz spectral excitation. Similar to the above, both pulses show relatively good agreement with the GPS design. However, in the gradient ramp regions where the pulse is 0 in the conventional design, the GPS design shows noise‐like characteristics (Figure 4 zoom‐ins) of similar amplitude throughout the pulse. Nonetheless, the profile simulations of the GPS design and conventional design agree well for both 0 and 440 Hz spectral excitation.

#### 4.1.4 2D selective RF pulse

**FIGURE 5:**
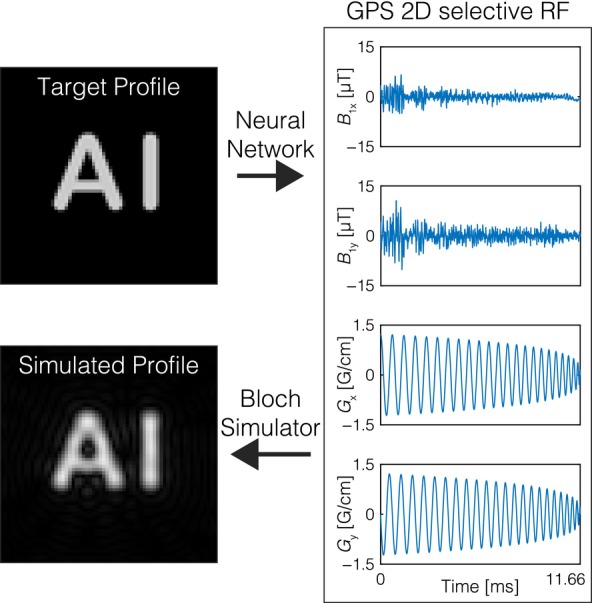
A 2D target profile composed of the letters “AI” (top left) was input as a target profile to the generalized RF pulse design using physics‐guided self‐supervised learning (GPS) framework, which designed a 2D selective RF pulse (top right) using predefined spiral trajectory gradients (bottom right) designed to meet scanner gradient specifications. The simulated 2D profile obtained from Bloch simulations (bottom left) exhibits some blur because of the limited extent to which excitation *k*‐space is sampled, but otherwise agrees well with the target profile.

The 2D target profile, GPS‐designed 2D selective RF pulse, and corresponding 2D simulation profile are shown in Figure 5. Comparing the 2D pulse's simulated profile with the target profile, the simulated profile exhibits some blur, which is because of the limited extent to which excitation *k*‐space is sampled. Nevertheless, both profiles agree well. Considering the variable density spiral trajectory used and good profile agreement indicates that GPS also considers density compensation during the design process.

### 4.2 Experiments

#### 4.2.1 Phantom

The 1D selective profile obtained using GPS design and SLR is presented in Figure 2A Experiment. In both passband and stopband regions, the phantom experiment results show excellent agreement and further concur with the simulation. $B_{1}$‐insensitive profiles obtained using GPS design and HS1 are shown in Figure 2B Experiment. The profiles from both pulses agree well over the entire range of applied $B_{1}^{\max}$, both showing $B_{1}$ insensitivity for $B_{1}^{\max}$ values beyond 18.8 μT, in accordance with simulations.

Phantom images obtained using spectral‐spatial selective pulses from GPS design and conventional design is shown in the right column of Figure 4 for both 0 (water) and 440 Hz (oil) spectral excitation. For 0 Hz spectral excitation, both pulses produce good spectral selectivity that shows good agreement with one another. This can be seen by the bright water bath in the image (Figure 4A), where the mineral oil vials appear dark, indicating that the water bath is excited, whereas the mineral oil vials are not. The same applies to spectral selectivity for 440 Hz. In both GPS design and conventional design, the vials filled with mineral oil appear bright, whereas the water bath appears dark (Figure 4B), indicating this time that the mineral oil vials are excited, but the water bath is not.

#### 4.2.2 In vivo

**FIGURE 6:**
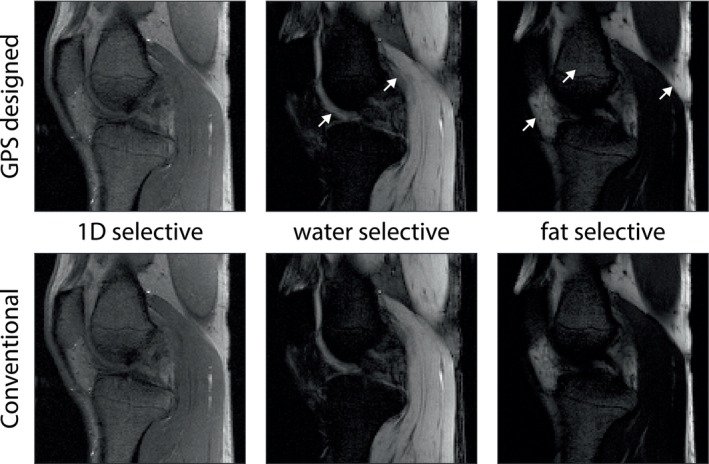
Anatomical knee images acquired using generalized RF pulse design using physics‐guided self‐supervised learning (GPS) 1D selective pulse (left column top) and Shinnar‐Le Roux (SLR) (left column bottom), spectral‐spatial (SPSP) selective pulse for 0 Hz (center column) and 440 Hz (right column) using both GPS and conventional. For the 1D selective images, both GPS and SLR agree well, with no noticeable difference. For 0 Hz water selective SPSP (center column), in both GPS (top row) and conventional (bottom row) images, the muscle and cartilage, indicated by arrows, appear bright, whereas bone marrow, infrapatellar fat pad, and other fatty tissue are dark, demonstrating that both pulse designs achieve good spectral selectivity at 0 Hz. The same can be said for the 440 Hz fat selective case (right column), whereby in both GPS (top row) and conventional (bottom row) images, bone marrow, infrapatellar fat pad, and other fatty tissue, indicated by arrows, appear bright whereas the muscle and cartilage appear dark.

Anatomical knee images acquired using GPS 1D selective pulse and SLR, SPSP selective pulse for 0 Hz (water) and 440 Hz (fat) using both GPS and conventional designs are presented in Figure 6. For the 1D selective images (Figure 6 left column), both GPS and SLR agree well with no noticeable difference, corroborating with both simulations and phantom experiments. For 0 Hz water selective SPSP (Figure 6 center column), in both GPS and conventional images, the muscle and cartilage appear bright, whereas the bone marrow, infrapatellar fat pad, and other fatty tissue are dark. This demonstrates that both pulse designs achieve good spectral selectivity at 0 Hz, agreeing well with each other with no noticeable difference and further supporting simulation and phantom experiment results. The same can be said for the 440 Hz fat selective case (Figure 6 right column), whereby in both GPS and conventional images, the bone marrow, infrapatellar fat pad, and other fatty tissue appear bright whereas the muscle and cartilage appear dark.

#### 4.2.3 Online adaptation

**FIGURE 7:**
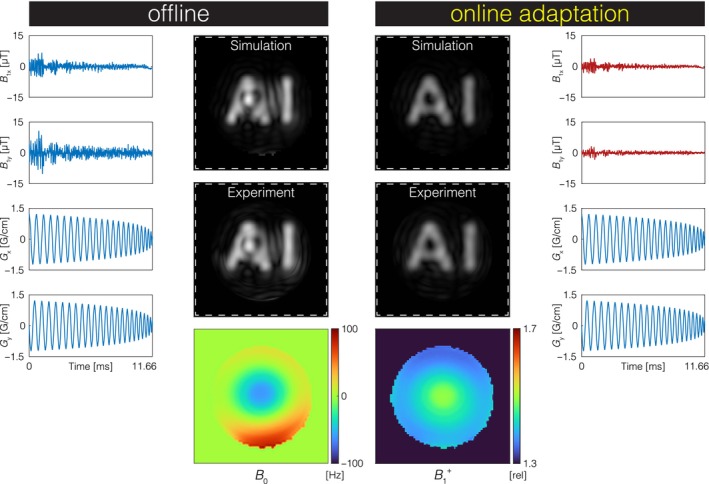
Phantom $B_{0}$/$B_{1}^{+}$ inhomogeneity online adaptation results. The left is the offline generalized RF pulse design using physics‐guided self‐supervised learning (GPS)‐designed 2D selective RF pulse and its corresponding excitation profile in the presence $B_{0}$/$B_{1}^{+}$ inhomogeneity. Experiment results show that the letters “A” and “I” are not well resolved, which was also confirmed by Bloch simulations. The online adapted GPS‐designed 2D selective RF pulse and its corresponding excitation profile are shown on the right. Compared with the offline design, the online adapted RF pulse has a lower overall amplitude, which reflects the high relative values of the $B_{1}^{+}$ map it has adapted to. Experiment results show both letters are now well resolved, which was also verified through simulations.

Phantom $B_{0}$/$B_{1}^{+}$ inhomogeneity online adaptation results are shown in Figure 7. The left half of this subfigure shows the offline GPS‐designed 2D selective RF pulse and its corresponding excitation profile in the presence $B_{0}$/$B_{1}^{+}$ inhomogeneity. The inner triangle region of the letter “A” is not well resolved, appearing bright in the middle instead of dark throughout. There are also two visible line streaks between the letters “A” and “I.” In addition, the bottom right portion of the phantom exhibits an increased streak signature, roughly correlating with the increase in $B_{0}$ inhomogeneity in this region. The agreement between Bloch simulations obtained with these $B_{0}$/$B_{1}^{+}$ maps included further confirm this. The online adapted GPS‐designed 2D selective RF pulse and its corresponding excitation profile can be seen in the right half of the subfigure. Compared with the offline design, the online adapted RF pulse has a lower overall amplitude, which reflects the high relative values of the $B_{1}^{+}$ map it has adapted to. Furthermore, both letters are now well resolved, with the previous bright spot of the inner triangle region of the letter “A” and visible line streaks between letters “A” and “I” both removed. This was also confirmed through simulations, which showed good agreement with the experiment.

**FIGURE 8:**
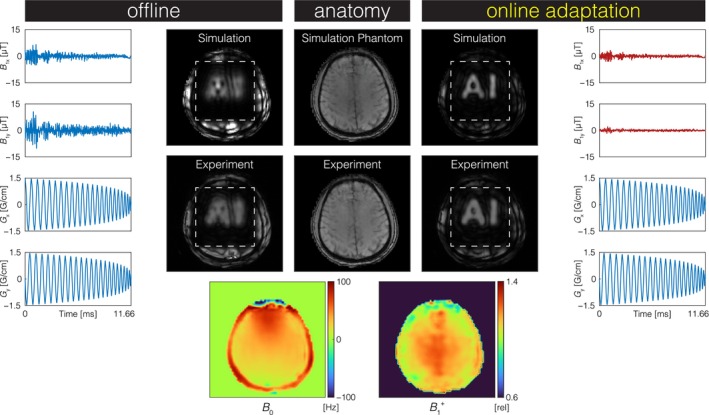
In vivo $B_{0}$/$B_{1}^{+}$ inhomogeneity online adaptation results. Offline generalized RF pulse design using physics‐guided self‐supervised learning (GPS)‐designed 2D selective RF pulse and its corresponding excitation profile in the presence $B_{0}$/$B_{1}^{+}$ inhomogeneity (left). Focusing within the dotted box, similar to the phantom results, the letters “A” and “I” are not well resolved, concurring with Bloch simulations. The online adapted GPS‐designed 2D selective RF pulse and its corresponding excitation profile are shown on the right. As was the case for the phantom, the online adapted RF pulse has a lower overall amplitude in comparison with the offline design, reflecting the high relative values of the $B_{1}^{+}$ map it has adapted to. Both letters are now well resolved, also confirmed by and showing good agreement with simulations. The existence of excitation outside the dotted box region is because of the 2D target profile provided as input to GPS being confined to the region within the dotted box, and the center anatomical image is displayed for reference.

In vivo $B_{0}$/$B_{1}^{+}$ inhomogeneity online adaptation results are shown in Figure 8. The left half of this figure shows the offline GPS‐designed 2D selective RF pulse and its corresponding excitation profile in the presence $B_{0}$/$B_{1}^{+}$ inhomogeneity. Focusing within the dotted box, similar to the phantom results, the inner triangle region of the letter “A” is not well resolved with three dark spots surrounding a bright middle instead of being dark throughout. A visible streak is also exhibited between the letters “A” and “I.” In addition, there is ringing near the top region of A, which corresponds to regions of high $B_{0}$ inhomogeneity. The existence of excitation outside the dotted box region is because of the 2D target profile provided as input to GPS being confined to the region within the dotted box. The good agreement between Bloch simulations obtained with these $B_{0}$/$B_{1}^{+}$ maps included further confirms this. The center anatomical image is shown for reference. The online adapted GPS‐designed 2D selective RF pulse and its corresponding excitation profile can be seen in the right half of the figure. As was the case for the phantom, the online adapted RF pulse has a lower overall amplitude in comparison with the offline design, reflecting the high relative values of the $B_{1}^{+}$ map it has adapted to. Both letters are now well resolved, with the previous three dark spots surrounding the bright middle in the inner triangle region of the letter “A” and visible line streaks between letters “A” and “I” both removed. This was also confirmed through simulations, showing good agreement. In all in vivo simulations, the obtained anatomical image with its intensity adjusted for $B_{1}^{+}$ inhomogeneity was used to accurately simulate.

## 5 DISCUSSION AND CONCLUSION

We have presented GPS, a self‐supervised learning framework that integrates a physics model to guide and enforce the learning process for MRI RF pulse design. As a proof‐of‐concept, we used the Bloch equations as the physics model and demonstrated its generalizability for designing a variety of RF pulses, which conventionally require separate dedicated algorithms. In addition, we showcased compensation for $B_{0}$/$B_{1}^{+}$ inhomogeneity using online adaptation in both phantom and in vivo experiments, further demonstrating GPS's flexibility and versatility in adapting to system imperfections in real time.

Despite showing similarities with conventional design, GPS‐designed RF pulses exhibit unique features, worth further exploration. We realized the differences in three aspects of our exemplified results. First, in terms of the design process, GPS designs an RF pulse that adheres to the boundaries of the target profile provided. For the 1D selective RF pulse design, the profiles resulting from GPS‐designed pulses using different target profile frequency ranges ([−4.096 kHz, 4.096 kHz] vs. [−8.192 kHz, 8.912 kHz] vs. [−16.384 kHz, 16.384 kHz] vs. [−32.768 kHz, 32.768 kHz]) show excellent agreement within their respective target frequency range. However, in their respective stopband regions outside their target ranges, the profile can differ in its non‐periodic randomly fluctuating signature (Figure S1). Second, the adherence to the boundaries of the 2D input target profile forGPS‐designed $B_{1}$‐insensitive RF pulses, which is the profile frequency and $B_{1}^{\max}$ range for which it is defined in, equally applies. However, the dynamics in which $B_{1}$‐insensitivity is accomplished is significantly different from the conventional method. In the conventional method, the time‐dependence of the amplitude and frequency modulation functions are designed to meet the adiabatic condition ($K\left(B_{1}^{\max},t\right)=\left|\frac{\gamma B_{1}^{\mathrm{eff}}\left(B_{1}^{\max},t\right)}{\dot{\alpha }}\right|>1$),^31^ which is the ratio between the effective magnetic field ($B_{1}^{\mathrm{eff}}\left(B_{1}^{\max},t\right)$) and its rate of change of orientation ($\dot{\alpha }$),throughout its duration. Comparing the adiabaticity of the HS1 and GPS‐designed $B_{1}$‐insensitive pulse (Figure S2), the HS1 pulse conforms to the adiabatic condition throughout its duration for both isochromats. However, for the GPS design, the adiabatic condition is violated for 62% of the duration. This indicates that instead of using the properties of adiabaticity, the physics module of the GPS framework guides the neural network module to invoke other mechanisms to achieve $B_{1}$‐insensitivity. A similar result was reported in another study,^18^ which used an RL approach to design $B_{1}$‐insensitive RF pulses. Finally, as was noted in the Results section, the GPS‐designed SPSP pulse was not 0 during the gradient ramp regions as in the conventional design, but instead showed random fluctuations (Figure 4 zoom‐in). Sampling of the RF pulse during the gradient ramp regions can further be exploited to decrease the peak power of the SPSP pulse for a given target flip angle. For example, SPSP pulses can be designed with GPS using regularization of $B_{1}^{\max}$. This necessitates the pulse to leverage the gradient ramp regions during the design process to lower $B_{1}^{\max}$ as shown in Figure S3, which decreases $B_{1}^{\max}$ by 15% compared to the non‐regularized design in achieving identical flip angle. Despite these unique differences, in practice, one would use a digitally designed target excitation profile as input to the GPS framework as was done in the 2D selective case. This has also been demonstrated for the 1D selective case where a rectangle function was used for the target excitation profile (Figure S4). Compared to the excitation profile obtained using an SLR pulse of identical pulse width and time‐bandwidth, the passband exhibited minimum ripples with a narrower transition width. Overall, this new RF pulse design approach through physics‐guided self‐supervised learning opens a new avenue to investigate RF pulse mechanisms that might not be observable in conventional RF design, which might possess great potential for achieving new design purposes.

The input–output structure of the GPS framework is different compared to other DL RF pulse design methods whereby the input and output are the same RF excitation profiles. Previous supervised methods rely on a broad input and corresponding output dataset such as diverse 2D excitation profiles with corresponding 2D RF pulses,^14^ limiting the type of RF pulse the method is able to design. Although unsupervised methods relieve the requirement of having an output dataset for the corresponding input,^17^ this approach nonetheless requires a broad input dataset, which influences the scope of the type of RF pulse design that is achievable. In GPS, the target learning objective RF pulse is generated at an intermediatory step and input into the Bloch simulator, which is used as a means for self‐supervision. Therefore, a case‐specific training strategy is used, which forgoes the need of a diverse training dataset and instead trains independently for each excitation profile, allowing for a more general RF pulse design approach. Whereas overfitting is avoided in dataset‐based learning methods because it can lead to a network that does not generalize well, in GPS, overfitting is beneficial because it results in an RF pulse profile that better matches the target input.

The computational efficiency of our algorithm is ensured through two mechanisms: rapid Bloch simulation through JIT and online adaptation through low‐rank network learning. The Bloch simulator used in this study is an iterative solution to Bloch equations, which recursively applies matrix rotation operations for each time step of the RF pulse. This leads to a long chain for backpropagation, which increases computation. We addressed computational efficiency by converting and running the PyTorch Bloch simulator module in C++ through JIT, which significantly accelerates the computation time for each iteration. Furthermore, to expedite the online adaptation, we transferred and fixed the pretrained parameters from offline learning and only updated the LoRA^29^ instead of tuning all network parameters. This is based on the assumption that a change of a smaller subset of low‐rank parameters can effectively encapsulate the necessary adjustments for a new task. As shown in Table 2, a 1D pulse design can be completed in minutes, and a more complicated 2D pulse requires tens of minutes to complete during offline learning. With both JIT and LoRA, we demonstrated that 2D RF pulse online adaptation can be completed in 4 min to produce dramatic improvement in profile quality by compensating for $B_{0}$/$B_{1}^{+}$ inhomogeneity. Although this is an encouraging result to show the potential of GPS pulse for imaging applications that are less scan time constrained, more research is needed to further improve the time efficiency for offline training and, more importantly, online adaption in time‐sensitive applications. Some plausible solutions worth deep investigation in this regard are to optimize the implementation of algorithms such as by using adjoint methods (e.g., neural ordinary differential equation)^41^ to further accelerate the long chain operation in the Bloch simulation, or to better leverage computational hardware architecture such as distributed GPU computation for parallel computing of gradient backpropagation.^42^ Finally, considering this online adaption of GPS pulse as a system calibration step, management of imaging protocol is also subject to future research where care must be cast to optimize the protocol workflow to avoid unnecessary idle time and improve the overall workflow efficiency.

To conclude, we have introduced a physics‐guided self‐supervised learning approach to design a variety of RF pulses. We further demonstrated its applicability to adjust for experimental imperfections such as $B_{0}$/$B_{1}^{+}$ inhomogeneity. GPS has great potential to become a general method for RF pulse design that can be practically applicable to real scan applications.

## Supporting information


**Figure S1.** Profiles of GPS 1D selective RF pulse designed with target profile frequency ranges [−4.096 kHz, 4.096 kHz] (blue), [−8.192 kHz, 8.912 kHz] (orange), [−16.384 kHz, 16.384 kHz] (yellow) and [−32.768 kHz, 32.768 kHz] (purple). Each pulse design shows excellent agreement within their respective target frequency range.
**Figure S2.** Adiabaticity of the HS1 and GPS‐designed *B*_1_‐insensitive pulse.
**Figure S3.** Sampling of the RF pulse during the gradient ramp regions can further be exploited to decrease the peak power of the SPSP pulse for a given target flip angle.
**Figure S4.** GPS‐designed 1D selective RF pulse amplitude and phase designed using a rectangular function as target profile are shown on the left. Simulation and phantom experiment results are displayed on the right half. Comparing the simulation results with SLR of identical specification, the GPS‐designed 1D selective pulse exhibits passband ripples 0.1% vs. 0.9%, stopband ripples 2% vs. 1.2%, and transition width 400 vs. 430 Hz. The rectangle function target profile is depicted by the black dashed line in the simulation plot.
