# Supplementary material for: Physics‐guided self‐supervised learning: Demonstration for generalized RF pulse design
Source: Magn Reson Med. 2024 Oct 9;93(2):657–72. doi: 10.1002/mrm.30307 (PMC11604838; doi:10.1002/mrm.30307)
Supplement: Supplementary file 1 — Figure S1. Profiles of GPS 1D selective RF pulse designed with target profile frequency ranges [−4.096 kHz, 4.096 kHz] (blue), [−8.192 kHz, 8.912 kHz] (orange), [−16.384 kHz, 16.384 kHz] (yellow) and [−32.768 kHz, 32.768 kHz] (purple). Each pulse design shows excellent agreement within their respective target frequency range. Figure S2. Adiabaticity of the HS1 and GPS‐designed B 1‐insensitive pulse. Figure S3. Sampling of the RF pulse during the gradient ramp regions can further be exploited to decrease the peak power of the SPSP pulse for a given target flip angle. Figure S4. GPS‐designed 1D selective RF pulse amplitude and phase designed using a rectangular function as target profile are shown on the left. Simulation and phantom experiment results are displayed on the right half. Comparing the simulation results with SLR of identical specification, the GPS‐designed 1D selective pulse exhibits passband ripples 0.1% vs. 0.9%, stopband ripples 2% vs. 1.2%, and transition width 400 vs. 430 Hz. The rectangle function target profile is depicted by the black dashed line in the simulation plot. [file MRM-93-657-s001.docx]

**Physics-Guided Self-Supervised Learning: Demonstration for Generalized RF Pulse Design**

**Supplementary Information**

GPS 1D selective RF pulse design – simulation study on different target frequency ranges

GPS 1D selective RF pulse design was carried out for target profile frequency ranges [-4.096 kHz, 4.096 kHz], [-8.192 kHz, 8.912 kHz], [-16.384 kHz, 16.384 kHz] and [-32.768 kHz, 32.768 kHz]. The figure below shows that each pulse design shows excellent agreement within its target frequency range. However, in its respective stopband regions outside its target ranges, instead of being flat, the profile differs in its non-periodic randomly fluctuating signature.


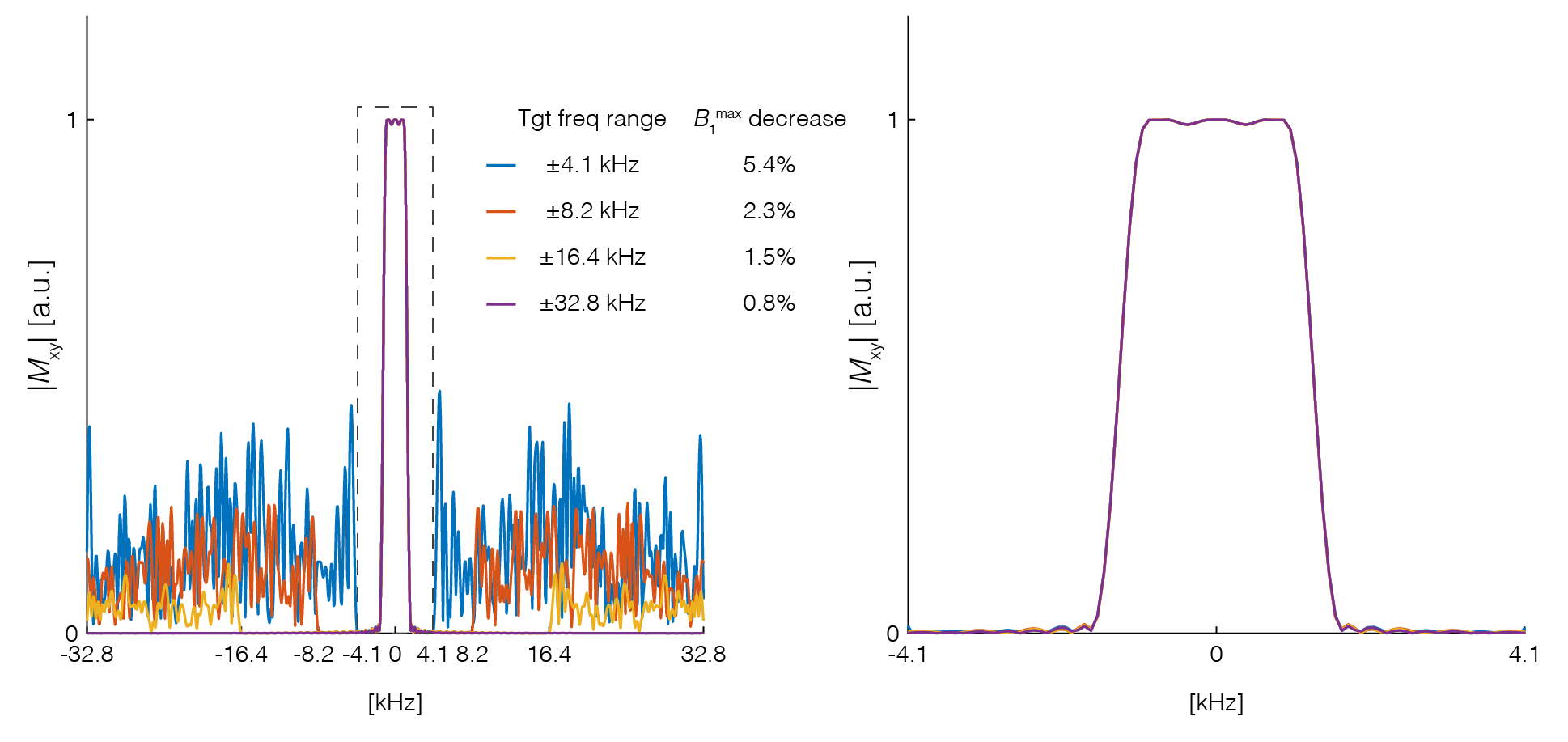


**Supporting Figure S1.** Profiles of GPS 1D selective RF pulse designed with target profile frequency ranges [-4.096 kHz, 4.096 kHz] (blue), [-8.192 kHz, 8.912 kHz] (orange), [-16.384 kHz, 16.384 kHz] (yellow) and [-32.768 kHz, 32.768 kHz] (purple). Each pulse design shows excellent agreement within their respective target frequency range.

GPS designed *B*_1_-insensitive pulse – adiabaticity study

The adiabaticity was calculated for both the HS1 and GPS-designed *B*_1_-insensitive pulse. As shown in the figure below, comparing the adiabaticity of the HS1 and GPS-designed *B*_1_-insensitive pulse, the HS1 pulse conforms to the adiabatic condition throughout its duration. However, for the GPS design, the adiabatic condition is violated for 62% of the duration. This indicates that instead of using the properties of adiabaticity, the physics module of the GPS framework guides the neural network module to invoke other mechanisms to achieve *B*_1_-insensitivity.


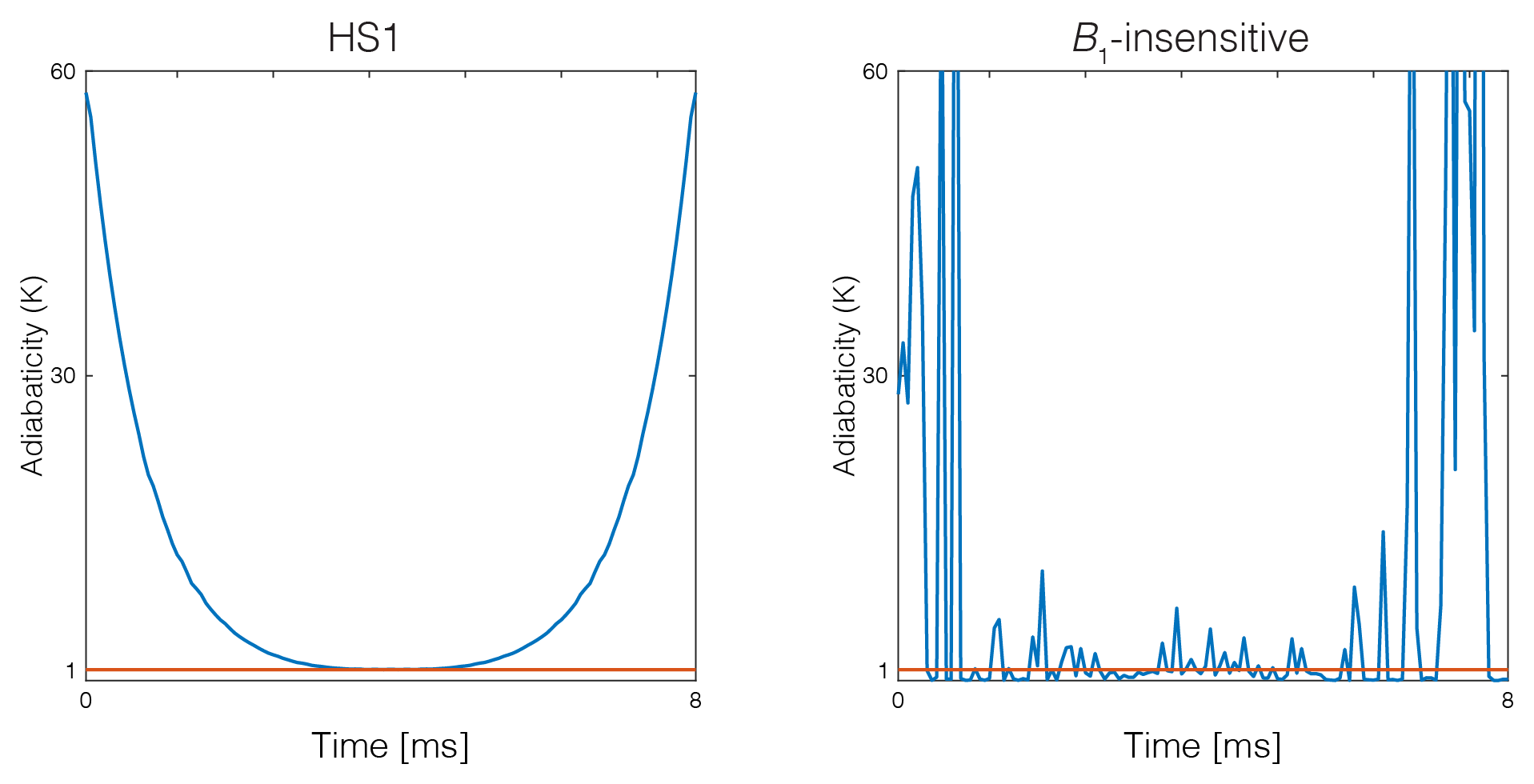


**Supporting Figure S2.** Adiabaticity of the HS1 and GPS-designed *B*_1_-insensitive pulse.

GPS SPSP pulse – designing with *B*_1_^max^ regularization study

The GPS-designed SPSP pulse was not 0 during the gradient ramp regions as in the conventional design but instead showed random fluctuations. Sampling of the RF pulse during the gradient ramp regions can further be exploited to decrease the peak power of the SPSP pulse for a given target flip angle. Here, we designed SPSP pulses with GPS using the regularization of $B_{1}^{\max}$. As can be seen in the figure below, comparing the pulses designed with and without $B_{1}^{\max}$, the regularized pulse shows greater fluctuation amplitude in the gradient ramp regions, indicating its utilization for lowering $B_{1}^{\max}$ by 15% compared to the non-regularized design in achieving an identical flip angle. The resulting profiles of both pulses show excellent agreement (Supplementary Figure S3).


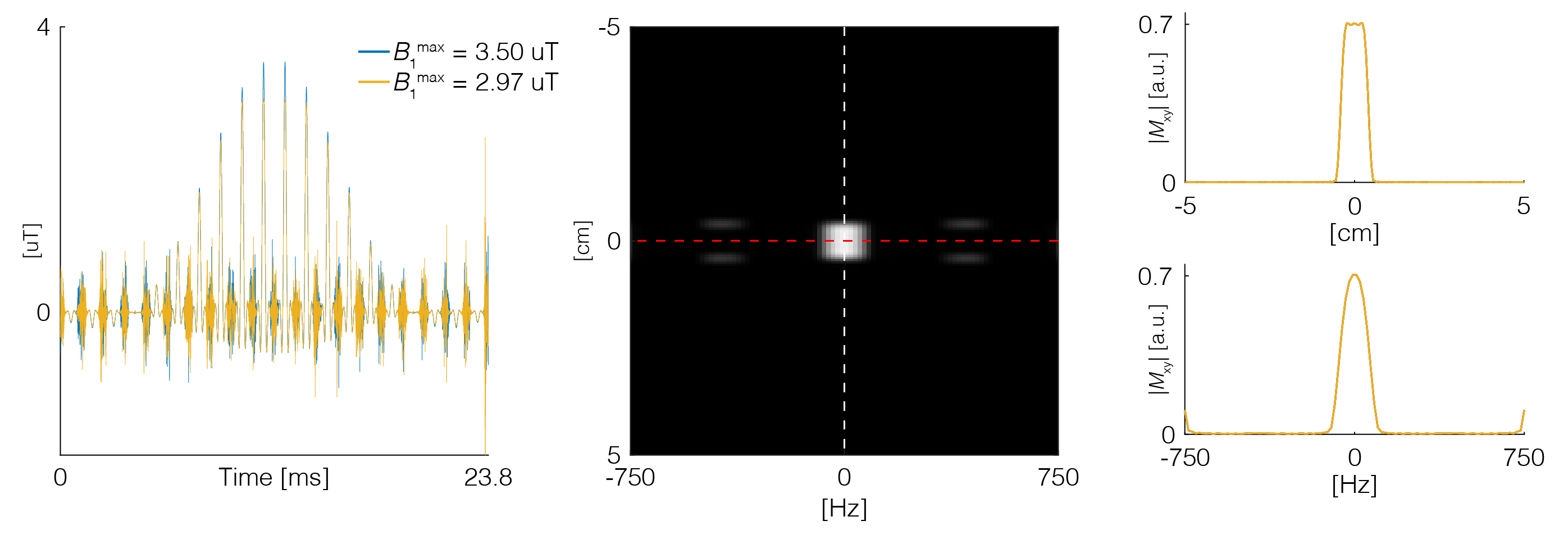


**Supporting Figure S3.** Sampling of the RF pulse during the gradient ramp regions can further be exploited to decrease the peak power of the SPSP pulse for a given target flip angle.

GPS 1D selective RF pulse designed with rectangular function target input profile

The rectangular function $M_{0}\Pi\left( \frac{f}{\mathrm{BW}} \right)=\left\{ \begin{matrix} 0 \mathrm{if} \left| f \right|>\frac{\mathrm{BW}}{2} \\ \frac{1}{2}M_{0} \mathrm{if} \left| f \right|=\frac{\mathrm{BW}}{2} \\ M_{0} \mathrm{if} \left| f \right|<\frac{\mathrm{BW}}{2} \end{matrix} \right.$, where $f$ $\left( =\gamma\vec{G}\cdot\vec{r} \right)$ is frequency and $\mathrm{BW}$ is excitation bandwidth, was used as input target excitation profile to the GPS framework for 1D selective RF pulse design. The design pulse width and time-bandwidth were set to 2.56 ms and 6.6, respectively. Comparing with SLR of identical pulse width and time-bandwidth, the GPS-designed 1D selective pulse exhibits passband ripples 0.1% vs. 0.9%, stopband ripples 2% vs. 1.2%, and transition width 400 Hz vs. 430 Hz, where the transition band was calculated using 0.1$M_{0}$ as stopband edge and 0.9$M_{0}$ as passband edge. The GPS-designed RF pulse, simulation and experiment results are shown below.


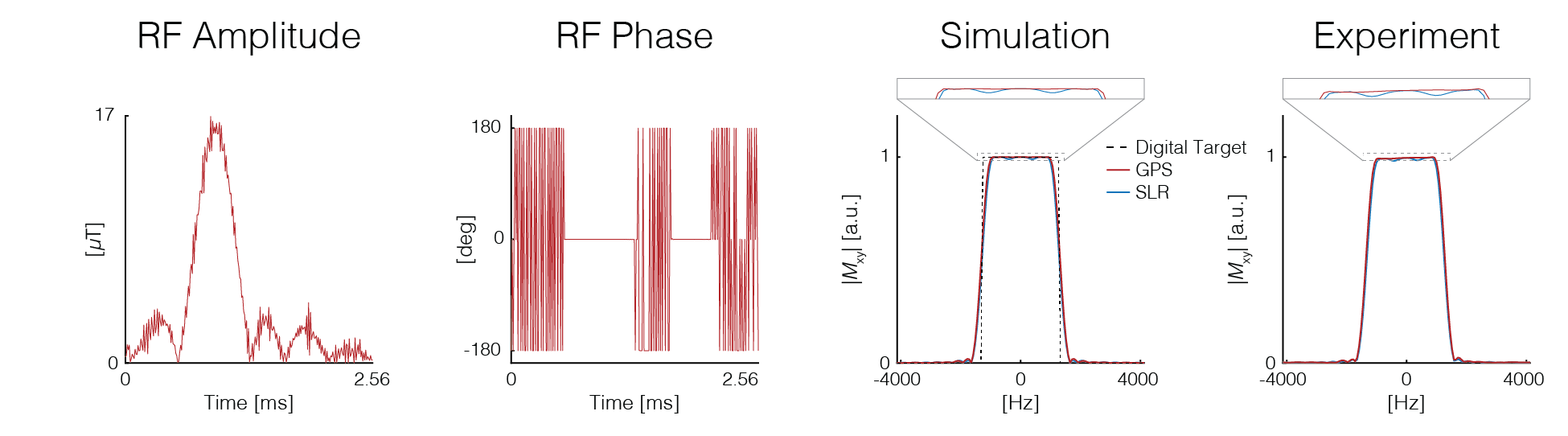


**Supporting Figure S4.** GPS-designed 1D selective RF pulse amplitude and phase designed using a rectangular function as target profile are shown on the left. Simulation and phantom experiment results are displayed on the right half. Comparing the simulation results with SLR of identical specification, the GPS-designed 1D selective pulse exhibits passband ripples 0.1% vs. 0.9%, stopband ripples 2% vs. 1.2%, and transition width 400 Hz vs. 430 Hz. The rectangle function target profile is depicted by the black dashed line in the simulation plot.
